# Analysis of Gene Expression Signatures in Cancer-Associated Stroma from Canine Mammary Tumours Reveals Molecular Homology to Human Breast Carcinomas

**DOI:** 10.3390/ijms18051101

**Published:** 2017-05-20

**Authors:** Julia Ettlin, Elena Clementi, Parisa Amini, Alexandra Malbon, Enni Markkanen

**Affiliations:** 1 Institute of Veterinary Pharmacology and Toxicology, Vetsuisse Faculty, University of Zürich, Winterthurerstr. 260, 8057 Zürich, Switzerland; julia.ettlin@uzh.ch (J.E.); elena.clementi@vetpharm.uzh.ch (E.C.); parisa.amini@vetpharm.uzh.ch (P.A.); 2 Institute of Veterinary Pathology, Vetsuisse Faculty, University of Zürich, Winterthurerstr. 268, 8057 Zürich, Switzerland; alexandra.malbon@uzh.ch

**Keywords:** cancer, dog, tumour, mammary carcinoma, tumour stroma, gene expression, cancer-associated stroma, tumour microenvironment

## Abstract

Cancer-associated stroma (CAS) plays a key role in cancer initiation and progression. Spontaneously occurring canine mammary carcinomas are viewed as excellent models of human breast carcinomas. Considering the importance of CAS for human cancer, it likely plays a central role in canine tumours as well. So far, however, canine CAS lacks characterisation, and it remains unclear whether the biology between CAS from canine and human tumours is comparable. In this proof-of-principle study, using laser-capture microdissection, we isolated CAS and normal stroma from 13 formalin-fixed paraffin embedded canine simple mammary carcinomas and analysed the expression of seven known human CAS markers by RT-qPCR (Reverse Transcription quantitative PCR) and validated some targets by immunohistochemistry. We found that Col1a1 (Collagen1α1), αSMA (alpha Smooth Muscle Actin), FAP (Fibroblast activation protein), PDGFRβ (Platelet-derived growth factor receptor beta), and Caveolin-1 were significantly upregulated in canine CAS, and the expression of CXCL12 (Stromal cell derived factor 1) significantly decreased, whereas MMP2 (Matrix Metalloproteinase 1) and IL6 (Interleukin 6) did not change. Our results suggest strong similarities in CAS biology in canine and human mammary carcinomas but also reveal some differences. To the best of our knowledge, this is the first report to provide a comprehensive expression analysis of the most important CAS markers in canine simple mammary carcinomas and further supports the validity of the dog as model for human cancer.

## 1. Introduction

The majority of all cancers are of epithelial origin and derive from a corrupted epithelial cell population that gives rise to aggressively growing tumour cells. However, these epithelial tumour cells are not living in an isolated environment, and, far from being self-sufficient, heavily depend on their microenvironment for growth and survival (reviewed in [1]). While the vast majority of research in the past has focused on the neoplastic cells themselves, recent progress has started to unveil the central importance of the tumour microenvironment in cancer formation and progression. The so-called cancer stroma consists of an extracellular matrix as well as a variety of cells, including endothelial cells, immune cells, and fibroblasts (reviewed in [2]). Under physiological conditions, stroma serves as an important barrier to prevent epithelial transformation (reviewed in [3]). However, in response to emerging epithelial cancerous lesions, the stromal compartment undergoes a reprogramming towards a tumour-supportive function, termed cancer-associated stroma (CAS), and plays a key role in cancer initiation and progression [1]. The pivotal role of CAS in many human carcinomas (such as breast, lung, prostate, and colorectal carcinomas) has been widely documented [4]. It has even been suggested that components of CAS serve as actual drivers, initiating the development of a tumour from precancerous cells (e.g., [2]). Abundant literature shows that CAS directly supports the growth of tumour cells through secretion and/or activation of cytokines, growth factors, nutrients, and proteases (e.g., reviewed in [1,4]). Studies performed in human clinical tumour samples have begun to shed light on mechanisms driving the formation of CAS as well as the molecular dialogue between CAS and tumour cells (e.g., [5,6,7,8]).

Due to the closely related pathophysiology, naturally occurring cancers in the domestic dog are progressively leveraged as a valuable source of information to better understand the biology behind tumour development and possibly find novel anti-cancer treatments [9,10,11]. Indeed, the study of canine cancer overcomes several of the limitations of genetically modified or xenograft rodent models for tumours [12,13]. Canine mammary tumours in particular are viewed as excellent models for human breast cancer due to strong clinical and molecular similarities and also availability of specimens [13,14]. In dogs, the majority of mammary cancer cases are classified either as simple carcinomas or as complex carcinomas [15]. Histologically, canine simple carcinomas very closely replicate the biology of human simple carcinomas (e.g., reviewed in [14]). Even at the molecular level, canine simple carcinomas replicate the genomic aberrations found in the human counterpart and have thus been demonstrated to faithfully represent human breast carcinoma [13]. Finally, canine mammary tumours are highly relevant in the veterinary clinical setting due to their incidence, as the most frequent cancer in intact female dogs, as well as the difficulties of therapeutic intervention (such as e.g., control of metastases and development of resistance to therapy) that are associated with all current cancer treatments [16].

Given the importance of CAS for the biology of human cancer, it likely plays a central role in the development and growth of canine tumours as well. So far, however, canine CAS greatly lacks characterisation, and it remains completely unclear whether similar markers are expressed in canine and human CAS. Therefore it remains unclear whether CAS has a role in the growth of canine tumours, what mechanisms are involved in its formation, and if canine CAS is comparable to human CAS. For these reasons, we set out to analyse the expression of several genes known to be expressed in the CAS of human breast tumours in formalin-fixed paraffin embedded (FFPE) specimens from canine simple mammary carcinoma cases. Our results provide a comprehensive overview, the first of its kind to our knowledge, of the expression of the most important CAS markers in dogs and suggest that CAS-related biology is very comparable between canine and human breast carcinoma.

## 2. Results

### 2.1. Selection of Cases Included in the Study

The main aims of our study were, firstly, to characterise the expression of known CAS-associated targets from human cancer in canine mammary tumour associated stroma and, secondly, to understand if large aspects of the underlying biology of CAS could be compared between dog and human breast cancer. To this end, we chose to include only canine simple carcinoma cases, as this type of tumour is recognised as a very close clinical, histological, and molecular correlate of human simple breast carcinoma [13,14]. Cases with obvious inflammation were excluded to avoid introducing unnecessary variability (for details, see Materials and Methods). With these criteria in mind (also see the Materials and Methods section), we selected a total of 13 canine simple mammary carcinomas, as defined by a board-certified veterinary pathologist (A.M.), from the archives to be included in the analysis. The mean age of the dogs at sample collection was 9.8 years, and further characteristics of patients and tumour samples can be found in Table 1.

### 2.2. Selection of Cancer Associated Stroma Markers to be Analysed in This Study

One of the inherent challenges in identifying CAS is that, because of its cellular and molecular heterogeneity, there is no single molecule that could specifically and reliably differentiate CAS from normal stroma. However, several studies performed with human breast cancer material have reported a variety of factors that are produced by CAS, the expression of some of which is correlated with a clinically unfavourable outcome. Using the available literature for gene expression changes in human breast cancer stroma, we selected targets that have been reported by several studies as typical CAS markers and represent a variety of different classes of molecules (Table 2).

### 2.3. Isolation of mRNA from Tumour Stroma and Matched Normal Stroma from Patient Material

**Figure 1:**
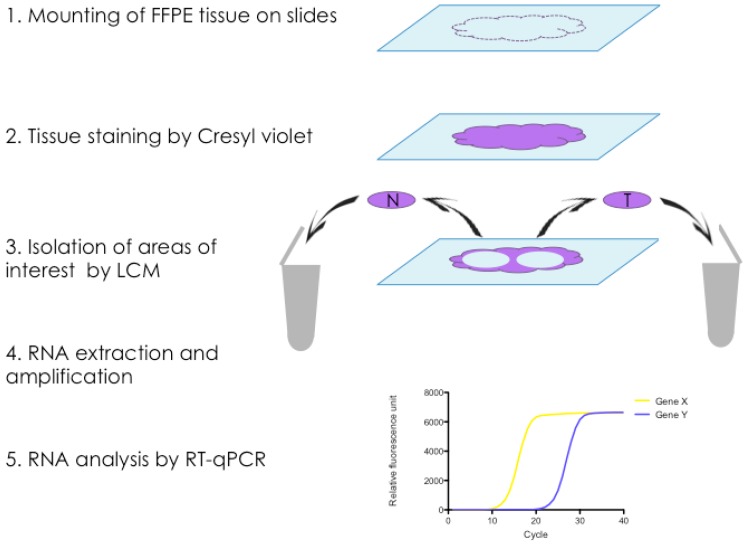
Protocol for isolation and analysis of matched normal and cancer-associated stroma from FFPE tissue sections. (1) Formalin-fixed paraffin-embedded (FFPE) tissue sections are cut and mounted onto PEN (Polyethylene naphthalate) Membrane Glass Slides (Applied Biosystems™); (**2**) Tissue is stained with Cresyl violet to facilitate visualisation of areas of interest under the microscope; (**3**) Areas of normal stroma (N) and tumour stroma (T) are isolated from the same slide under the microscope using Laser-Capture Microdissection, and harvested into separate tubes; (**4**) RNA extraction, quality control, quantitation, and preamplification; (**5**) Relative mRNA levels of selected genes are analysed by RT-qPCR.

**Figure 2:**
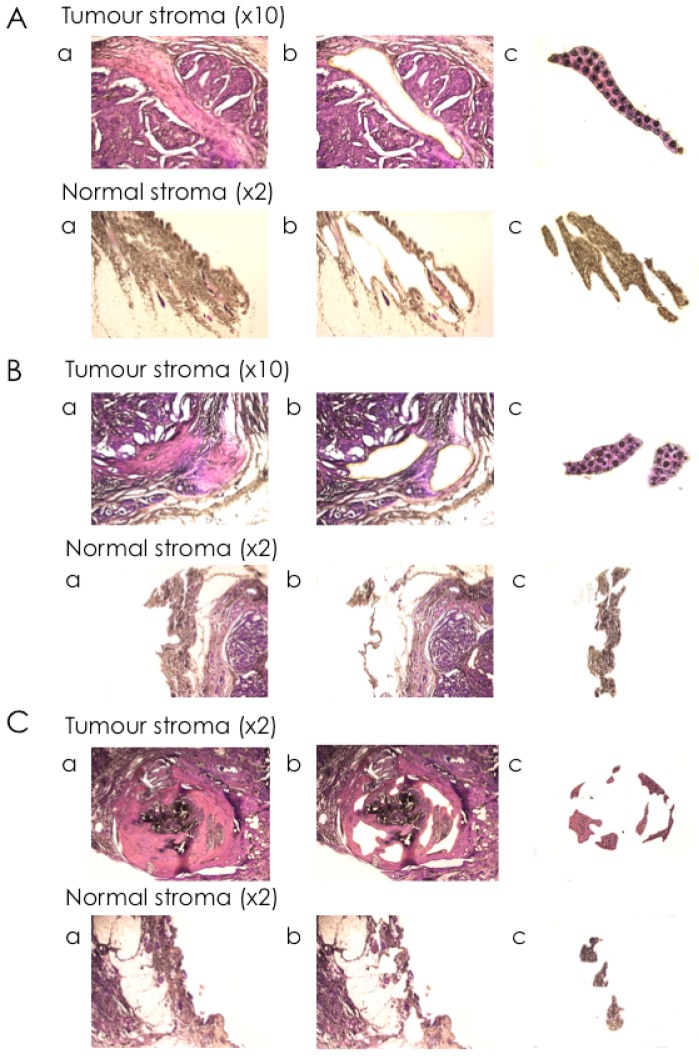
Selective isolation of cancer-associated stroma and normal stroma from canine simple mammary carcinomas by laser-capture microdissection. Representative images of tissue mounted on the slide were taken at ×2 and ×10 magnification, as indicated, (**a**) before dissection; (**b**) after dissection; and (**c**) of the CapSure^®^ Cap containing the excised tissue sections to validate the isolation of the selected cells. The dark spots visible in the CapSure^®^ Cap samples denote the area of the melted thermoplastic film of the cap by the infrared laser and thus of adhering tissue. Three representative cases were chosen. Cases: (**A**): Case #3; (**B**): Case #4; (**C**): Case #6.

To specifically isolate RNA from CAS and normal stroma from FFPE tissue sections of clinical mammary carcinoma cases, we established a protocol for laser-capture microdissection (LCM), a technique that allows the precise excision of areas of interest from microscopic tissue sections, for canine FFPE tissue sections, followed by RNA isolation (Figure 1). Using the ArcturusXT™ Laser Capture Microdissection System (ThermoFisher Scientific, Waltham, MA, USA), we isolated matched normal stroma and CAS from 13 clinical cases of simple mammary carcinoma (Table 1). Importantly, normal stroma and CAS were both isolated from the exact same tissue section to minimise differences in tissue quality and processing, thus allowing for optimal comparability of the two correlates. The areas of interest were defined by a board-certified veterinary pathologist (Alexandra Malbon, A.M.), and microscopic validation of the tissue before and after excision ensured the selective isolation of CAS and normal stroma (Figure 2). RNA isolation, analysis, and preamplification were performed as specified in the Materials and Methods section. As expected for FFPE tissue samples [41], the analysis of mRNA quality and quantity revealed highly fragmented RNA and low yields (Table S1). Nevertheless, the mRNA was amenable to analysis by RT-qPCR.

### 2.4. Expression Analysis of Cancer Associated Stroma Markers by RT-qPCR

**Figure 3:**
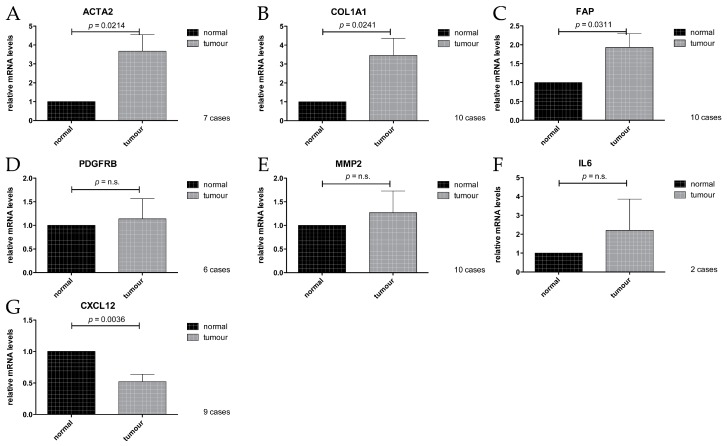
Relative mRNA levels of CAS-associated genes in normal stroma and tumour stroma isolated by laser-capture microdissection (LCM), measured by RT-qPCR. (**A**): ACTA2; (**B**): COL1A1; (**C**): FAP; (**D**): PDGFRB; (**E**): MMP2; (**F**): IL6; (**G**): CXCL12. Values are mean values ±SEM, normalised to expression levels in normal stroma. *p*-values were calculated using student’s *t*-test, and significance was set at *p* ≤ 0.05. n.s. = not significant.

To analyse expression of the targets of interest (Table 2) in the RNA isolated from the FFPE tissue sections, RT-qPCR was performed, comparing CAS with the respective matching normal stroma. Three cases had to be excluded from the analysis due to insufficient housekeeping gene performance, probably due to low RNA abundance. PDGFRB, ACTA2, and CXCL12, as well as IL6, could not be reliably amplified in all cases, leading to different numbers of cases that were analysed for each gene, as indicated in the respective panels (Figure 3). For details on which cases yielded data for which primers, see Table S1. 

We found that the mRNA levels of ACTA2, COL1A1, and FAP were all significantly increased in CAS compared to the normal stroma (Figure 3A–C). These findings are consistent with data obtained from studies on human material (Table 2 and references therein) and validate our approach to specifically isolate RNA from CAS and normal stroma from canine FFPE mammary carcinoma samples. No significant difference in mRNA expression levels could be detected for PDGFRB or MMP2 (Figure 3D,E), possibly due to a combination of a small sample size and relatively modest changes in expression of the gene, or perhaps simply because they might not change. Interestingly, the levels of CXCL12 were significantly lower in CAS compared to normal stroma (Figure 3), which is in contrast to the literature analysing human samples (Table 2 and references therein). The reasons for this discrepancy are currently unclear but might suggest a difference in the role of CXCL12 in stroma of canine compared to human mammary carcinomas. Taken together, our results showed that at least COL1A1, ACTA2, and FAP were significantly upregulated on mRNA levels in CAS from canine mammary carcinoma similar to human mammary carcinoma samples, whereas CXCL12 is downregulated. These data suggest that the underlying biology of CAS is highly comparable, at least in some aspects, between dogs and humans and that COL1A1, ACTA2, and FAP can be used as markers of CAS in canine mammary carcinomas.

### 2.5. Expression Analysis of Cancer Associated Stroma Markers by Immunohistochemistry

In order to validate our mRNA expression findings (Figure 3), to confirm that the measured changes were derived specifically from cells deriving from the stromal compartment and not influenced by contamination by regions containing epithelial cancer cells, and to possibly still extract data from the cases that had not yielded mRNA data, immunohistochemical (IHC) analysis of all thirteen cases was performed for αSMA (alpha Smooth Muscle Actin, the product of *ACTA2* gene), FAP (Fibroblast Activation Protein), PDGFRβ (Platelet-derived Growth Factor Receptor beta), MMP2 (Matrix Metalloproteinase 1), and SDF1 (Stromal Derived Factor 1, the product of *CXCL12* gene) (Figure 3). Additionally, we analysed the expression of Caveolin-1 (Cav1), a protein that is sometimes shown to be increased in CAS but, if decreased in CAS, has been shown to predict early recurrence and poor clinical outcome in human breast cancer (e.g., reviewed in [42]), and FGF2, another marker upregulated in CAS [2,39].

**Figure 4:**
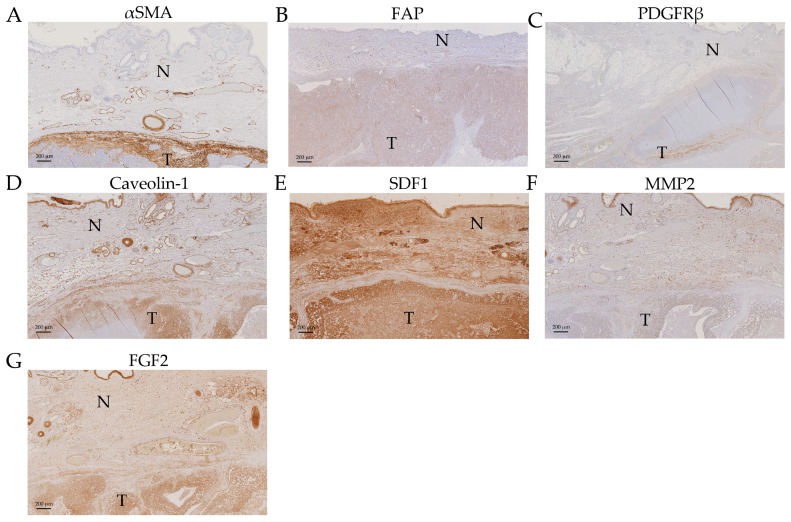
Immunohistochemical staining of a canine simple mammary carcinoma sample. Images are from Case #6), except for FAP, for which the image was taken from case #5. Pictures were taken at ×2 (for PDGFRβ) and ×5 (for the other gene products) magnification. T = tumour stroma, N = normal stroma. (**A**): αSMA; (**B**): FAP; (**C**): PDGFRβ; (**D**): Caveolin-1; (**E**): SDF1; (**F**): MMP2; and (**G**): FGF2.

**Figure 5:**
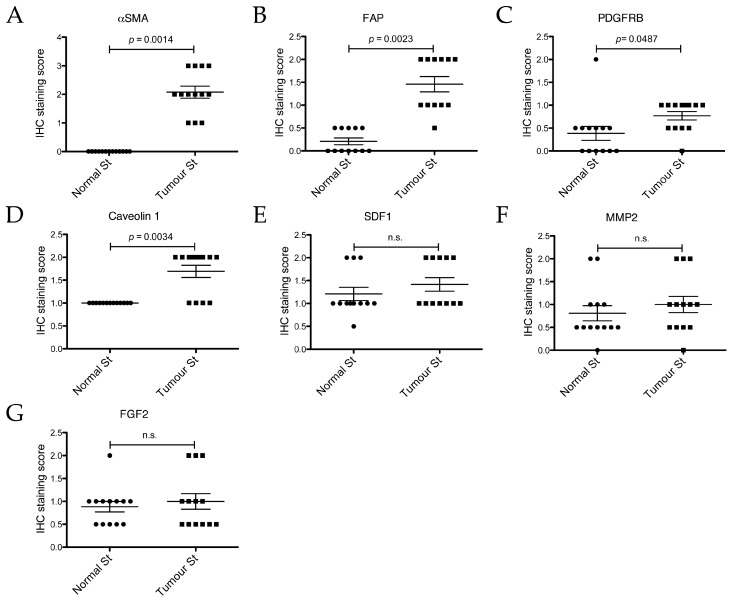
Immunohistochemical staining scores of the normal and tumour stroma (normal st and tumour st, respectively) from the 13 different cases for different proteins. Slides were stained with the indicated antibodies and scored. (**A**): αSMA; (**B**): FAP; (**C**): PDGFRβ; (**D**): Caveolin 1; (**E**): SDF1; (**F**): MMP2; (**G**): FGF2. IHC for FAP could only be performed on 12 cases due to the unavailability of case #7 at the time of processing. Plotted are all individual values and the mean ± SEM of the IHC staining scores. *p*-values are two-tailed and were calculated using the Wilcoxon signed rank test, with significance set at *p* ≤ 0.05. n.s. = not significant.

Immunohistochemically stained tumour sections were scored by a board-certified veterinary pathologist (A.M.), according to the following score-system: 0 = negative, 0.5 = negligible, 1 = mild, 2 = moderate, 3 = strong (see also Materials and Methods). We found αSMA, FAP, PDGFRβ, and Caveolin-1 to be significantly upregulated in CAS compared to normal stroma (Figure 4A–D and Figure 5A–D).

Staining for αSMA was only detectable in CAS as well as all vessel walls, whereas normal stroma or epithelial cancer cells remained negative (Figure 4A), and the IHC staining score was significantly higher in CAS compared to normal stroma (Figure 5A). These findings are in accordance with the literature from human breast cancer studies showing an upregulation of αSMA in CAS (Table 2) and validate our RT-qPCR results (Figure 3A). Importantly, these results further underline the specificity of our isolation protocol for CAS and normal stroma.

Mild to moderate FAP staining could be clearly detected in the tumour stroma and was significantly stronger than in the normal stroma, where only negligible staining could be detected, except for vessel walls (Figure 4B, Figure 5B and Figure S1). Staining for FAP in epithelial cancer cells was mostly negligible. These findings are in accordance with our RT-qPCR data (Figure 3C) and with literature from human breast cancer studies that have demonstrated FAP upregulation in CAS (Table 2).

Staining for PDGFRβ was significantly more intense in CAS compared to normal stroma, and no staining could be detected in epithelial cancer cells (Figure 4C and Figure 5C). This suggested that even though no significant increase in PDGFRB mRNA levels using RT-qPCR could be detected (Figure 3D), possibly due to a combination of low sample size with small expression changes, protein levels of PDGFRβ increased in CAS, which is similar to findings from human breast cancer stroma (Table 2 and references therein).

Tissue analysed with Cav1 antibody showed mild to moderate staining for the tumour stroma and only mild staining for the normal stroma (Figure 4D). Additionally to CAS, other cell types such as neoplastic epithelial cells, myoepithelial cells lining tubules, smooth muscle cells, and cells of blood vessels showed positive staining with the Caveolin-1 antibody. The IHC staining score for Cav1 was significantly higher for CAS compared to normal stroma (Figure 5D).

While the RT-qPCR of CXCL12 mRNA levels showed a significant decrease in CAS (Figure 3G), analysis of SDF1 protein expression by IHC revealed no significant changes between CAS and normal stroma, while neoplastic epithelial cells stained strongly (Figure 4E and Figure 5E). The reason for the discrepancy between the RT-qPCR and the IHC results remains to be further investigated. One possible explanation for this could be that stabilisation of the protein via posttranslation modifications interferes with protein turnover, even though the transcription of the corresponding mRNA has been downregulated. Furthermore, MMP2 and FGF2 failed to show any significant differences in IHC staining scores between CAS and normal stroma (Figure 4F,G and Figure 5F,G). The fact that MMP2 staining intensity did not change significantly is in accordance with the result of RT-qPCR (Figure 3E). Thus, the results from our IHC studies show clearly that αSMA, FAP, PDGFRβ, and Cav1 are all significantly upregulated in canine CAS, largely mimicking the events known from human CAS.

In summary, this study provides, to the best of our knowledge, the first thorough analysis of the gene expression signatures of known CAS-related genes from human tumour samples by RT-qPCR and IHC in FFPE tissues from dog mammary carcinomas.

## 3. Discussion

Recent cancer research has expanded its focus from mutated cancer cells to their microenvironment since the importance of the tumour stroma in cancer initiation, progression, and metastasis and the development of adaptive resistance to therapies has been unveiled [1,43,44]. To reveal the biology of cancer, the dog represents an ideal model organism as canine cancer occurs spontaneously, shows similar clinical presentation, and overcomes some limitations of rodent models. So far however, there are only very limited data regarding the reprogramming of normal stroma into CAS in canine cancers compared to studies performed in human samples [12,13]. To expand the knowledge regarding the biology of CAS in canine cancers, we set out to investigate the expression of known human CAS markers in the tumour stroma of canine simple mammary carcinoma specimens. To do this, we established the isolation of CAS and matched normal stroma by laser-capture microdissection (LCM) using FFPE canine simple mammary carcinoma samples (Figure 1). So far, few studies have been performed using LCM for analysing FFPE tissue samples [45,46], whereas most studies were performed using fresh frozen tissue samples [21,47] since fresh-frozen specimens promise a higher yield and much increased quality of mRNA. Despite low quantity and quality of the extracted RNA, as expected from the literature (Table S1), we were able to analyse the expression of all selected target genes (Table 2) by RT-qPCR (Reverse Transcription quantitative PCR) (Figure 3). These analyses were complemented by immunohistochemical staining to validate the procedure as well as the obtained data and further extend our observations (Figure 4). In the following, the findings for each target are discussed in detail.

### 3.1. ACTA2/αSMA

The upregulation of ACTA2 in CAS of our canine mammary carcinoma samples is consistent with the results from human studies in which the increase of its gene product, αSMA, is associated with poor prognosis [29,30,31,32,33,34]. αSMA plays a role in cell motility as it is a major constituent of the cytoskeleton and is expressed by myofibroblasts during wound healing as well as cancer-associated fibroblasts (CAFs) [2]. Importantly, we could further validate our observations by an IHC analysis of αSMA protein levels, which were significantly upregulated in stromal cells of the tumour but not in normal stroma (Figure 3A, Figure 4A and Figure 5A). This finding is consistent with another report demonstrating an increase in aSMA immunoreactivity in stroma of canine simple mammary carcinomas [48]. The specific localisation of staining with αSMA to CAS but not normal stroma or neoplastic epithelial cells further underlined the specificity of our approach in isolating CAS and normal stroma by LCM.

### 3.2. COL1A1/Collagen 1

We found COL1A1 to be upregulated in the canine tumour stroma by RT-qPCR (Figure 3B). This gene encodes the Collagen1 alpha chain 1, which forms part of the extracellular matrix. This upregulation of COL1A1 in CAS is consistent with findings in human studies [22,23,24,25] and matches the finding that collagen is progressively deposited during breast cancer development resulting in increased tissue stiffness as a classical fibrosis-type response of neoplasms [49].

### 3.3. Fibroblast Activation Protein, FAP

FAP, a serine protease expressed in the reactive stromal fibroblasts of epithelial cancers and the granulation tissue of healing wounds, was also found significantly overexpressed in the CAS of our canine tumour specimens by RT-qPCR (Figure 3C). Importantly, IHC for FAP confirmed this upregulation on protein levels (Figure 4B and Figure 5B, Figure S1). An increase in FAP expression in canine mast cell tumour stroma by IHC has been recently demonstrated [50]. FAP is known to be overexpressed in CAS from human breast cancer as well [27,28], and has the capacity to degrade gelatin and type 1 collagen and therefore influences the remodelling of the ECM, supporting the formation of a tumour-permissive milieu [28,51].

### 3.4. PDGFRB/PDGFRβ

PDGFRB encodes a tyrosine kinase receptor of the platelet-derived growth factor (PDGF) family. This protein, PDGFRβ, is secreted by CAFs and triggers cancer growth and increased pericyte coverage of vessels, resulting in increased vessel function [18]. High stromal PDGFRβ is associated with metastasis, larger tumour size, high histopathological grade, and shorter survival in human breast cancer [17,18,19,51]. In our dataset, no significant change in relative mRNA levels comparing tumour and normal stroma was observed (Figure 3D). However, it is possible that the inability of our analysis to determine any significant change is caused by a combination of a small sample size and a moderate effect size, which would necessitate a larger dataset for reliable detection. Therefore, the six cases included in this analysis are not enough to give a proper assessment of the situation in canine mammary cancers. Therefore, more samples would need to be analysed in order to draw a valid conclusion for the mRNA levels of PDGFRβ in canine mammary cancer stroma. Indeed, by IHC staining, we detected a statistically significant increase in staining of the tumour stroma compared to the normal stroma for PDGFRβ, indicating an increase in PDGFRβ protein in CAS (Figure 4C and Figure 5C). This finding is well in accordance with published data for human CAS [18].

### 3.5. CXCL12/SDF1

CXCL12 is a chemokine secreted by cancer associated myofibroblasts and binds to the CXCR4 receptor on epithelial cells, enhancing their proliferation, migration, and invasion and thus playing a role in tumorigenesis [20,33,35,43]. Moreover, CXCL12 is involved in angiogenesis by playing a part in the recruitment of endothelial progenitor cells into cancerous tissue [35,43]. Instead of being upregulated, as observed in many human studies [20,35,36], we found a downregulation of CXCL12 in the CAS of our specimens (Figure 3G). A lack of an adequate amount of myofibroblasts collected of the tumour stromal compartment compared to the normal stromal compartment seems not to be the main cause since ACTA2 and FAP are also expressed in myofibroblasts and show an opposing result (Figure 3A,C). Poor mRNA quality and an overall low amount of tissue samples may have had an influence on the validity and proper detection of CXCL12, or, quite simply, the canine isoform of CXCL12 may not play the same role in tumorigenesis in dogs as in humans. IHC staining for SDF1, the gene product of CXCL12, did not reveal differences between CAS and normal stroma in our analysis (Figure 4E and Figure 5E). This could be a consequence of the stabilisation of the protein via posttranslation modifications, which interferes with protein turnover, even though the transcription of the corresponding mRNA has been downregulated. Another explanation would be unspecific antibody crossreactivity with other targets that covers the real effect. Further analysis of a larger dataset as well as validation and extension of these findings are required to draw further conclusions.

### 3.6. MMP2

An analysis of MMP2 revealed no significant change in gene expression (Figure 3E) or protein levels by IHC (Figure 4F, Figure 5F), even though several breast cancer studies [20,21,22,23] have found an upregulation of MMP2 in the tumour stroma of human patients. MMP2 is a metalloproteinase secreted by CAFs and also tumour associated macrophages and functions by remodelling the ECM, resulting in tumour progression, invasion, and metastasis and the support of angiogenesis [21,43]. It is possible that a lack of increase of MMP2 expression in canine tumour stroma is due to lack of isolation of tumour associated macrophages in our case, as we sought to avoid areas of obvious inflammation when isolating stroma (see Materials and Methods). Moreover, CAFs may have not expressed high amounts of MMP2 since most CAS areas collected in this study were from areas inside the tumour mass and not at its border, where most of the remodelling processes are thought to take place. Malignant canine mammary tumours have been found to express higher levels of MMP2 than benign tumours or normal mammary tissue [52]. Importantly, the expression of MMP2 in these cases was detected in the myoepithelial cells lining the basement membrane of tubuloalveolar structures in benign tumours, while malignant tumours showed MMP2 expression in the neoplastic cells themselves. This study did not specify any expression of MMP2 in the stroma. Similarly, another report showed elevated levels of MMP2 expression by IHC and protein activity in neoplastic cells, with the highest levels in malignant tumours, whereas no differences in mRNA levels using the whole tumour could be found [53]. This study also found a higher level of MMP2 expression in the stroma of tumours, with the highest immunoreactivity found in the fibroblasts closest to the epithelial cells. Taking into account these results, it is possible that the cell layer closest to the tumours was not always excised during the isolation of CAS in order not to not risk a contamination of CAS material with neoplastic cells. If the protein was indeed produced in this cell layer, this would explain why we did not detect an increase in mRNA levels. Again, this needs to be further investigated in a larger cohort. Finally, it remains unclear why we were not able to detect an increase in MMP2 staining by IHC as demonstrated by [53], which could also be related to differences in the antibody used.

### 3.7. Interleukin 6, IL6

Examination of IL6 levels by RT-qPCR was successful for both tumour and normal stroma in only two cases and therefore clearly showed no significant difference in gene expression, which precludes any further interpretation of these data (Figure 3F). The cytokine is primarily secreted during acute or chronic inflammation by inflammatory cells or CAFs promoting tumorigenesis, angiogenesis. and metastasis [51]. Low amounts of tissue and bad mRNA quality may have had an influence on the proper detection of IL6 as well as the avoidance of inflammatory areas during tissue collection (see Materials and Methods section), which contain most of the IL6 secreting cells. Indeed, the study of Chavey et al. [37] showed a correlation between the number of tumour associated macrophages and IL6 levels in breast cancer, supporting this hypothesis.

### 3.8. Caveolin-1

Caveolin-1 is known to be expressed in cancer-associated fibroblasts, myoepithelial cells underlying the luminal epithelial cells, endothelial cells, and adipocytes, which clearly correlates with our detection (Figure 4D, Figure 5D) [54,55]. While, in humans, a decrease in expression of Cav1 in tumour stroma is generally considered to be a marker of poor prognosis [42], increases in Cav1 in CAS have also been documented to be associated with higher tumour aggressiveness [56]. Data on Cav1 expression in CAS from canine mammary tumours is less clear, mainly because studies have mostly focused on staining intensities in the tumour epithelia themselves and not in CAS particularly (e.g., [55]). Further studies are warranted for a more thorough analysis and an evaluation of the significance of these results.

Concluding, our data show that canine CAS shows similar changes in key CAS-molecules to those found in human cancer samples, suggesting that the underlying biology is very comparable. This further validates the use of canine simple mammary carcinomas as a model for human breast cancer [9,10,12,13,57]. Furthermore, it is possible that some of these changes in canine CAS render the cancer similarly more aggressive in its malignant behaviour and that such changes are associated with poor prognosis, as is the case with human cancers [51]. Unfortunately however, as our dataset is yet too small and there are no survival data available, these questions can not be addressed at this point in time.

Since difficulties with primer performance in a subset of samples reduced cases that could be included in the statistical analysis, analysis of more samples is needed to further validate our findings and unveil more reliable and more subtle changes. It is likely that next-generation RNA sequencing technologies would yield much more and precise data from these samples. Though technically challenging with such small amounts of highly degraded RNA, the establishment of this approach is expected to yield much more valuable information regarding the biology of CAS in dogs, to further our understanding of tumour biology, and perhaps lead to biomarker discovery and the development of new therapies for human and canine mammary carcinomas.

## 4. Materials and Methods

### 4.1. Selection of Cases for LCM

Thirteen dog mammary carcinoma samples were provided by the Institute of Veterinary Pathology of the Vetsuisse Faculty, Zürich. All of the samples were formalin-fixed, paraffin-embedded tissue samples from either the Small Animal Hospital of Zurich or external cases sent in by veterinarians practising in Switzerland. Cases were selected with the help of a board-certified veterinary pathologist (A.M.) using the following criteria; female dogs, simple mammary carcinomas, appropriate tumour stroma content, FFPE samples not older than two years [58], areas with no obvious or only negligible inflammation, and samples were paraffin-embedded on their arrival day at the Pathology, (i.e., no prolonged storage in formalin). During our initial screening for suitable cases, those which contained only highly inflamed stroma were excluded. In selecting areas for LCM from our chosen cases, no regions with aggregates of inflammatory cells were included. The cells were not specifically counted per field as this would be highly variable according to the proportion of the field taken up by stroma. Rather, if only individualised leukocytes (i.e., single figures of most commonly lymphocytes and plasma cells) were present within a stromal region, it was considered appropriate to select and any areas with aggregated inflammatory cells were removed.

### 4.2. Tissue Processing and Staining

Biopsy samples had been fixed immediately in 10% neutral buffered formalin and subsequently routinely embedded in paraffin. For total mRNA analysis, formalin-fixed, paraffin-embedded tissue sections cut at 10 µm were used. Diethylpyrocarbonate (DEPC) treated water was used for the microtome HM 360 (ThermoFisher Scientific, Waltham, MA, USA), and the blade was cleaned with RNase away™ (ThermoFisher Scientific, Waltham, MA, USA). The tissue was mounted on PEN Membrane Glass Slides (Applied Biosystems™, Waltham, MA, USA). The mounted tissue sections were left to dry overnight at room temperature (http://support.moleculardevices.com). To visualise the areas of interest, the tissue sections were stained for initial scrape tests with H&E, and for all other interventions with Cresyl Fast Violet according to [59] with slight modifications (Table 3). The slides were completely air dried before microdissection to allow for proper excision performance. For every tissue sample that underwent LCM, a second tissue slide was stained with conventional Hematoxylin-Eosin staining to allow for validation of tissue morphology in case of uncertainty using the Cresy violet stain. The reagents used were xylene (Thommen-Furler AG, Rüti bei Büren, Switzerland), ethanol (Sigma-Aldrich, St. Louis, MO, USA), Hematoxylin Solution modified acc. to Gill II (Merck KGaA, Darmstadt, Germany), Ammonium Hydroxide Solution (Sigma-Aldrich), Cresyl Fast Violet (Fluka AG, Buchs, Switzerland), and DEPC treated water (Carl Roth, Karlsruhe, Germany).

### 4.3. Laser-Capture Microdissection (LCM)

Tumour grading (Table S2) was performed by a veterinary pathologist (A.M.), according to the grading system adapted for canine simple mammary carcinoma by Clemente et al. ([60] from an existing human grading system [61]. Before microdissection, the identification of tumour stroma in samples was performed by a veterinary pathologist (A.M.). The criteria for stroma were fibroblastic cells, endothelial cells and pericytes of small vessels, only single inflammatory cells to avoid areas with heavy inflammation, and no adipocytes. We also excluded medium or large vessels to maximize the fibrous portion of the sampled stroma. The chosen stroma was morphologically different to normal stroma, being more compact and frequently sclerotic. Using the criteria of previous papers (e.g., [21,47]), we ensured that “normal” was at least 2 mm away from the neoplasm, but in practice the overlying epidermis was almost always included in the biopsy so that the normal dermis immediately beneath was sampled whenever possible (excluding adnexal structures).

For microdissection, the ArcturusXT™ Laser Capture Microdissection System (Thermo Scientific) and Arcturus^®^ CapSure^®^ Macro LCM Caps (Life Technologies) were used. Highly enriched populations of normal or tumour-associated stroma from the specimen were identified and isolated according to the manufacturer’s protocol. Normal stroma samples were isolated from the same slides as tumour-associated stroma, from regions specified by a veterinary pathologist (A.M.) that presented no obvious alterations or were at least 2 mm away from the tumour [47]. The isolation of cells of interest was verified by microscopic examination of the LCM cap as well as the excised region after microdissection (Figure 2). After excision, the caps were put on 0.5 mL microcentrifuge tubes (Eppendorf^®^ Safe-Lock Tubes, Hamburg, Germany) and placed on ice until proceeding with mRNA isolation.

### 4.4. Isolation of mRNA from FFPE Tissue Sections Isolated by LCM

Extraction of mRNA was performed immediately after microdissection within three hours after staining, due to potential RNase contamination during storage [59]. A Recover All™ Total Nucleic Acid Isolation Kit for FFPE (Ambion™) was used to extract the mRNA according to the manufacturer’s protocol with the following small adjustments. As long exposure to xylene has been shown to be detrimental to mRNA integrity [62] and deparaffinisation by xylene had already been performed to stain the sections, the first deparaffinisation step using xylene and 100% ethanol was skipped, and the excised tissue was directly immersed in a 0.5 mL microcentrifuge tube containing 100 µL Digestion Buffer and 4 µL Protease. To get the tissue into the solution, a sterile blade and forceps were used to peel off the thermoplastic film on the cap containing the captured cells. The heating time and temperature in step C-2a were adjusted to 3 h at 50 °C, followed by 20 min at 70 °C, according to manufactures protocol “Optimized Extraction and Quantification of RNA from FFPE Samples for Gene Expression Analyses” (https://tools.thermofisher.com). To elute the mRNA from the column, RNase-free water was used to avoid the effects of the elution buffer on downstream applications. The eluate was aliquoted before analysis and stored at −80 °C. mRNA abundance and quality was analysed using the 4200 or 2200 Tape Station Software using the High Sensitivity RNA ScreenTape kit (Agilent Technologies, Santa Clara, CA, USA), according to the manufacturer’s protocol.

### 4.5. cDNA Synthesis and Preamplification

To retrotranscribe mRNA into cDNA, the iScript™ cDNA Synthesis Kit by Bio-Rad was used according to the manufacturer’s protocol, using a maximum of 15 µL of mRNA per reaction. This kit allows generation of cDNA with combination of oligo (dT) and random hexamer primers using low mRNA inputs and is optimized for fragments below 1 kb of length. cDNA preamplification was done using the TaqMan^®^ PreAmp Master Mix (2×) (Applied Biosystems™) to produce sufficient cDNA for qPCR analysis. The preamplification was performed according to the manufacturer’s protocol using 14 PCR cycles.

### 4.6. Reverse Transcription quantitative PCR

Quantitative real-time PCR (RT-qPCR) was performed using KAPA PROBE FAST qPCR Kit Master Mix (2×) Universal reagents (Kapa Biosystems, Wilmington, MA, USA), with 2.5 µL preamplified cDNA per reaction in a total volume of 10 µL. RT-qPCR reactions were run in duplicates on the CFX384 Touch™ Real-Time PCR detection system (BioRad, Hercules, CA, USA). The primers used in this study are detailed in Table 4. The comparative CT (Cycle threshold) method was applied for the quantification of gene expression, and the values were normalised against GAPDH, PPIA, and B2M as endogenous controls. The results were expressed as fold changes in mRNA levels of cancer-associated stroma over normal stroma. The primers were either customised Taqman^®^ gene expression assays specifically designed to detect the canine isoforms of the targeted genes (ThermoFisher Scientific), used at final concentrations of 900 nM primers and 250 nM probes, or, for canine GAPDH, purchased from Microsynth (Balgach, Switzerland) and used at a final concentration of 300 nM primers and 200 nM probe [63]. All primer pairs have been validated by the manufacturer, or in the case of GAPDH by [63], and displayed at approximately 100% amplification efficiency.

### 4.7. Graphical Display of Results and Statistical Analysis

For all statistical analysis and graphical displays, the program GraphPad Prism (www.graphpad.com) was used. The data was first tested for normality using the D’Agostino and Pearson omnibus normality test and the Shapiro-Wilk normality test. If the data followed a Gaussian distribution (normally distributed data), student’s *t*-test was performed to assess significance for a two-tailed *p*-value with α = 0.05. If data did not pass the normality test (non-normally distributed data), a Wilcoxon Signed Tank Test was performed to assess the significance for a two-tailed *p*-value with α = 0.05.

### 4.8. Immunohistochemistry

Formalin-fixed paraffin-embedded (FFPE) tissue sections (2 µm thickness) were mounted on positively charged slides and dried overnight at 37 °C. Drying was followed by the deparaffinisation of the slides with four xylene baths for 5 min each using the Tissue-Tek^®^ Prisma^®^ and Film^®^ (Sysmex, Horgen, Switzerland). For rehydration, a degressive alcohol series using 100% ethanol, 95% ethanol, 70% ethanol, and distilled water was performed. Slides used for SDF1 immunohistochemical staining underwent an antigen-retrieval pretreatment after rehydration by putting the slides into EDTA-buffer (basic buffer pH 9.0) and then into a pressure cooker for 20 min at 98 °C, followed by rinsing with distilled water. Thereafter all the sections were put in TBS wash-buffer 3006 (Dako, Carpinteria, CA, USA). Immunohistochemical staining for FAP was performed as specified in [50]. Staining for PDGFR-β, MMP-2, SDF1, FGF-2, and Caveolin-1 was performed with the Dako Autostainer (Agilent Technologies) using polyclonal rabbit antibodies (Table 5) overnight at 4 °C and for αSMA using monoclonal mouse antibody (Table 5) for 1h at room temperature. The antibodies were diluted in the dilution-buffer S2022 (Dako, Carpinteria, CA, USA). After incubation with the primary antibody, the slides were rinsed with TBS wash-buffer and blocked with peroxidase (peroxidase blocking buffer, Dako S2023) for 10 min at room temperature. The αSMA slides were treated with a link biotinylated secondary antibody for 15 min at room temperature and rinsed with TBS before peroxidase blocking. Next, the slides were rinsed with TBS and incubated with the EnVision™ + System HRP Rabbit Kit (Dako K4003) for 30 min at room temperature or, for αSMA, incubated with the Dako Real™ Detection Kit (Dako K5001, K5003) for 15 min at room temperature. Before removing the slides from the Autostainer, they were rinsed with TBS and incubated with DAB (diaminobenzidine) Dako K3468 (Dako, Carpinteria, CA, USA) for 10 min at room temperature. Removing the slides from the Autostainer, they were rinsed with distilled water and counterstained for 2 s in Hematoxylin (modified acc. to Gill II, Merck KGaA, Darmstadt, Germany). Finally the sections were rinsed with tap water, dehydrated in the Prisma^®^ machine (70% ethanol, 95% ethanol, 100% ethanol and xylene), and covered with the Tissue-Tek^®^-Film^®^.

The immunohistochemical staining was scored semi-quantitatively by a board-certified veterinary pathologist (A.M.) without prior knowledge of the PCR results. Scores were based on the section as a whole, e.g. if one region composing <25% stained strongly whilst the majority remained unstained, the score was given as mild. As the aim was to identify differences between groups, comparisons were performed between groups for a given antibody rather than between antibodies. The scoring system was therefore adapted slightly to suit the staining behaviour of the antibody and allow maximum discrimination between cases. When a stain showed scant variation in staining intensity (e.g., αSMA), the slides were assessed purely numerically. Where intensity was highly variable, the two were factored together. Therefore a sample with strong staining in <25% would score moderate, and if in two cases 90% of cells stained but one weakly and the other strongly, the latter would score strong and the former moderate. Furthermore, neoplastic epithelial cells were examined for positive staining to check if the selected genes were specifically expressed in stromal cells. The staining intensity was scored in a scale from 0 to 3 (0: negative, 0.5: negligible (rare individual cells/barely perceptible staining), 1: mild (up to 25% positively staining cells), 2: moderate (25–75% positively staining cells), 3: strong (>75% positively staining cells).

## Figures and Tables

**Table 1:** Overview of cases included in this study. Clinical data from dogs with simple mammary carcinoma; Case # = case number as referred to within this study; f/n = female, neutered; n.d. = not disclosed; age = age at excision of tumour; age of sample = time between initial tumour excision and sampling of stroma/RNA extraction.

Case #	Gender	Breed	Age (Years)	Subtype of Simple Carcinoma	Age of Sample (Months)
1	f	Basset	12	tubular	3
2	f	Vizsla	10	cystic-papillary	18
3	f	Samoyed	5	tubulo-papillary	7
4	f	Maltese	14	tubular	3
5	f	Tibetan Terrier	12	tubular	15
6	f/n	West Highland White Terrier	12	tubular-solid	13
7	f	Havanese	13	tubular	11
8	f	Chihuahua	8	tubulo-papillary	7
9	f/n	Bracke	9	cribriform	14
10	f/n	n.d.	13	tubular	7
11	f/n	Appenzell Mountain Dog	6	tubular	18
12	f	Boxer	9	tubulo-papillary	8
13	f	n.d.	4	cystic-papillary	23

**Table 2:** List of the cancer-associated stromal targets selected for analysis in this study. List of gene names and their respective protein names that were assessed by RT-qPCR (Reverse Transcription quantitative PCR), immunohisto–chemistry, or both. “Expression in CAS” summarises the expression trend as observed in human studies (see References). The column “qPCR” denotes which of the targets were analysed by RT-qPCR, whereas the column “HC” specifies which targets were assessed by immunohistochemistry. The “Ref.” column indicates selected references to either original publications or reviews. * For Cav1, expression studies are somewhat discordant, although generally a decreased Cav1 expression in CAS is associated with poor prognosis.

Gene Name	Protein Name	Function	Expression in CAS	qPCR	IHC	Ref.
*PDGFRB*	PDGFRβ (Platelet-derived growth factor beta)	Cell-surface tyrosine kinase receptor	upregulated	×	×	[17,18,19]
*MMP2*	MMP2 (Matrix metalloproteinase 2)	Metalloproteinase	upregulated	×	×	[20,21,22,23]
*COL1A1*	Col1a1 (Collagen 1 α 1)	Extracellular matrix	upregulated	×		[22,23,24,25,26]
*FAP*	FAP, Fibroblast activation protein	Serine protease	upregulated	×		[27,28]
*ACTA2*	αSMA (α smooth muscle actin, aorta)	Cytoskeleton	upregulated	×	×	[29,30,31,32,33,34]
*CXCL12*	SDF1 (Stromal cell-derived factor 1)	Chemokine	upregulated	×	×	[20,35,36]
*IL6*	IL-6 (Interleukin-6)	Cytokine	upregulated	×		[37,38]
*FGF2*	bFGF (Basic fibroblast growth factor)	Growth factor	upregulated		×	[2,39]
*CAV1*	Cav1 (Caveolin-1)	Possibly stabilisation of caveolar membranes	Downregulated *		×	[2,40]

**Table 3:** Staining protocol for Cresyl violet staining of formalin-fixed paraffin embedded (FFPE) tissue sections.

Cresyl Violet Staining for FFPE Tissue Sections	
100% Xylene, bath 1	5 min
100% Xylene, bath 2	5 min
100% Ethanol	30 s
95% Ethanol	30 s
70% Ethanol	30 s
dH_2_O	10 s
Cresyl violet (75% Ethanol with Diethylpyrocarbonate (DEPC) treated dH_2_O, pH 8.0)	15 s
dH_2_O	10 s
70% Ethanol	10 s
95% Ethanol, bath 1	10 s
95% Ethanol, bath 2	10 s
100% Ethanol, bath 1	30–60 s
100% Ethanol, bath 2	30–60 s

**Table 4:** List of primers used for RT-qPCR. The “c” before each gene indicates that primers were designed to detect the canine isoforms of the intended targets.

Gene Target	Sequence	Amplicon Length (nt)	Taqman^®^ Order Number or Reference
*cGAPDH*	Fw: 5′-GCTGCCAAATATGACGACATCA-3′ Rev: 5′-GTAGCCCAGGATGCCTTTGAG-3′ Probe: 5′-TCCCTCCGATGCCTGCTTCACTACCTT-3′	75	[63]
*cPPIA*	Manufacturer’s proprietary information	92	Cf03986523_gH
*cB2M*	Manufacturer’s proprietary information	87	Cf02659077_m1
*cPDGFRB*	Manufacturer’s proprietary information	60	Cf02626568_g1
*cMMP2*	Manufacturer’s proprietary information	58	Cf02623423_m1
*cCOL1A1*	Manufacturer’s proprietary information	87	Cf02623126_m1
*cFAP*	Manufacturer’s proprietary information	69	Cf02657429_m1
*cACTA2*	Manufacturer’s proprietary information	86	Cf02668774_mH
*cCXCL12*	Manufacturer’s proprietary information	86	Cf02625258_m1
*cIL6*	Manufacturer’s proprietary information	68	Cf02624151_m1

**Table 5:** Details of the primary antibodies used for Immunohistochemistry.

Antibody	Source, Order Information	Type	Dilution	Reference
PDGFR-β	Santa Cruz Biotechnology, sc-432	Rabbit polyclonal	1:50	[64]
FAP	Abcam, ab53066	Rabbit polyclonal	1:100	[50]
MMP-2	Thermo Scientific, Ab-7, #RB-1537-P1	Rabbit polyclonal	1:100	[53]
αSMA	Dako, Clone 1A4, Code M0851	Mouse monoclonal	1:400	-
SDF1	Abcam, ab9797	Rabbit polyclonal	1:100	-
FGF-2	Santa Cruz Biotechnology, (147) sc-79	Rabbit polyclonal	1:100	[65]
Caveolin-1	Santa Cruz Biotechnology, (N-20) sc-894	Rabbit polyclonal	1:100	[55]

## Supplementary Materials

Supplementary materials can be found at www.mdpi.com/1422-0067/18/5/1101/s1.
