# Supplementary material for: Analysis of Gene Expression Signatures in Cancer-Associated Stroma from Canine Mammary Tumours Reveals Molecular Homology to Human Breast Carcinomas
Source: Int J Mol Sci. 2017 May 20;18(5):1101. doi: 10.3390/ijms18051101 (PMC5455009; doi:10.3390/ijms18051101)
Supplement: Supplementary file 1 [file ijms-18-01101-s001.pdf]

Supplementary Materials: Analysis of Gene Expression Signatures in Cancer-Associated Stroma from Canine Mammary Tumours Reveals Molecular Homology to Human Breast Carcinomas

Julia Ettlin, Elena Clementi, Parisa Amini, Alexandra Malbon and Enni Markkanen

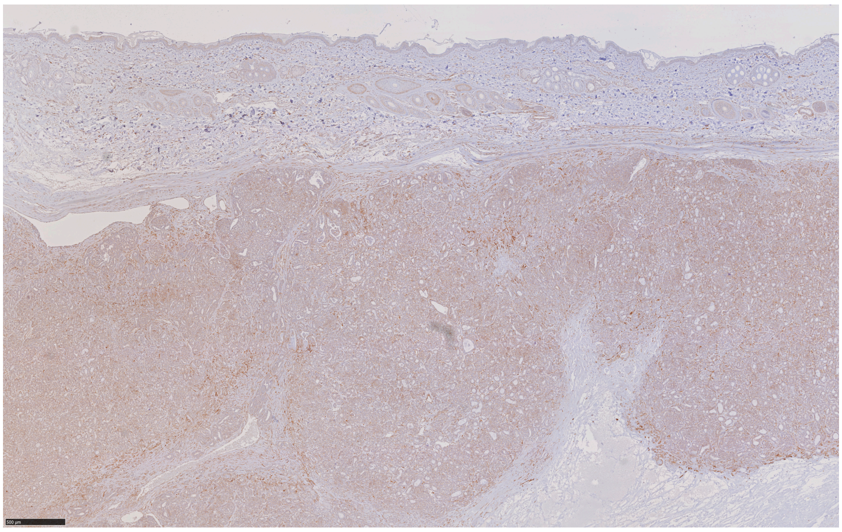

(A)

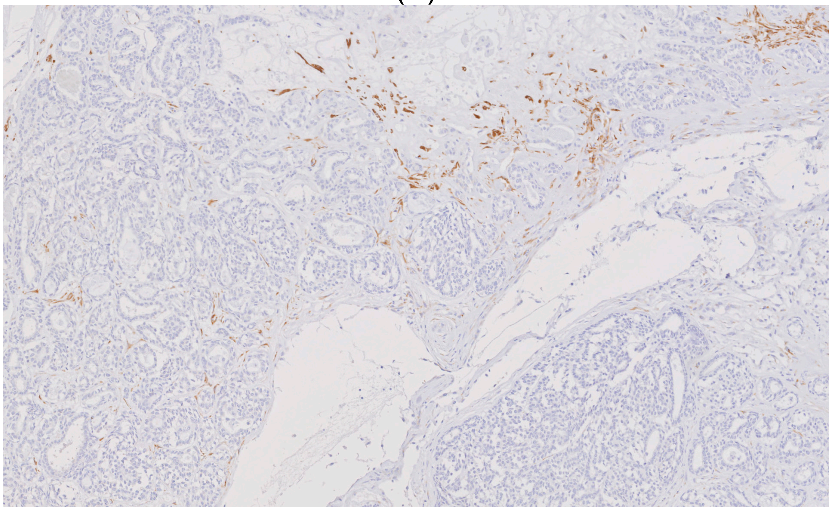

(B)

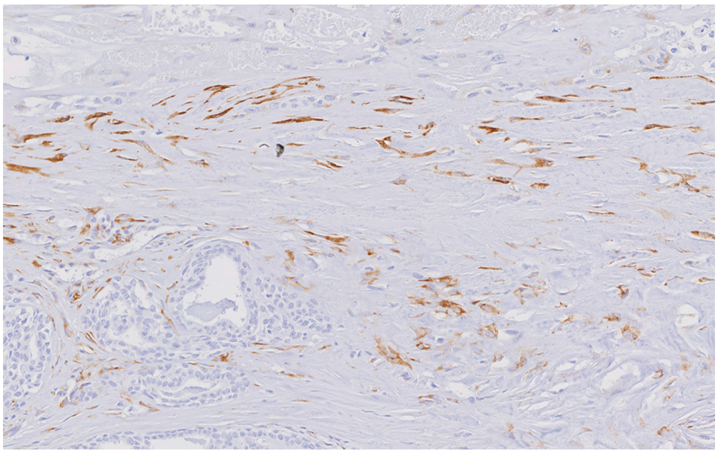

(C)

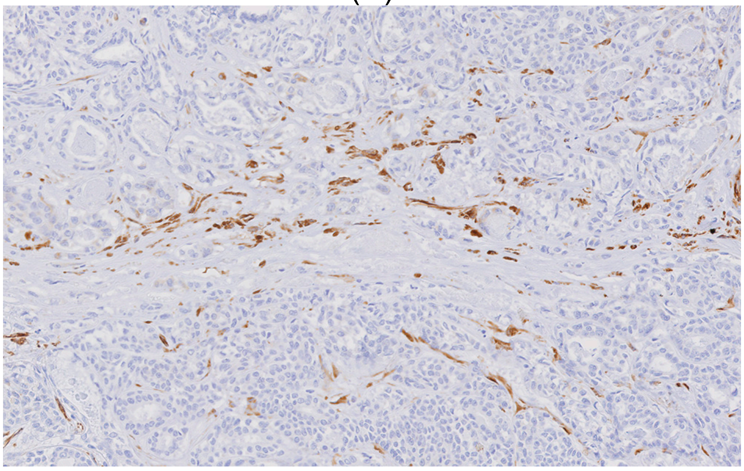

(D)

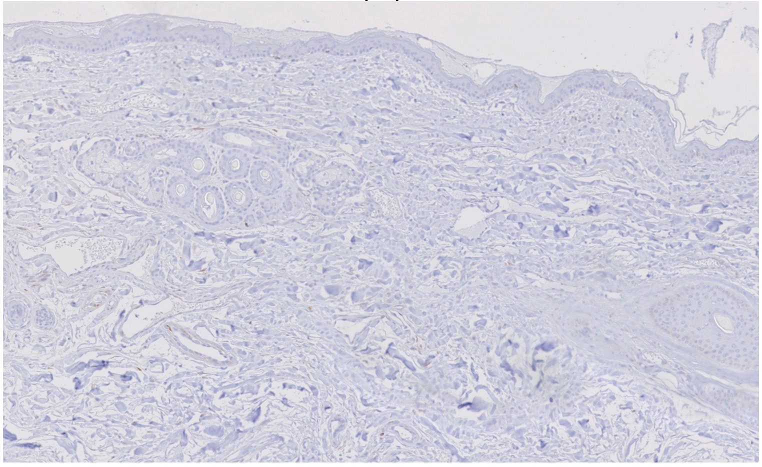

(E)

Figure S1. Additional IHC pictures for FAP staining for case#5**. A**: higher resolution image for FAP staining as shown in Figure 4B. **B-E:** higher magnification images for FAP staining with a higher dilution of the antibody (1:150). **B:** 10x magnification of tumour tissue showing a moderate staining score for FAP in CAS. **C** and **D:** 20x magnification of tumour tissue showing a moderate staining score for FAP in CAS. **E:** 5x magnification demonstrating that normal epithelia and normal stroma staining for FAP is negative or negligible.

Table S1. mRNA quality, total mRNA yield and qPCR performance of the LCM for the samples in this study. mRNA quality, defined by RNA integrity number (RIN), and total mRNA yield obtained from normal (N) and tumour (T) stroma for each clinical case. '-' denotes not measurable. Columns labelled ACTA2, COL1A1, FAP, PDGFRB, MMP2, IL6 and CXCL12 show which of the samples yielded quantifiable RT-qPCR data for the respective primers used (+).

<table>
<tr><th colspan="2">Clinical case #</th><th>RIN</th><th>Total mRNA yield [ng]</th><th>ACTA2</th><th>COL1A1</th><th>FAP</th><th>PDGFRB</th><th>MMP2</th><th>IL6</th><th>CXCL12</th></tr>
<tr><td rowspan="2">1</td><td>N</td><td>1.4</td><td>4.5</td><td rowspan="2">+</td><td rowspan="2">+</td><td rowspan="2">+</td><td rowspan="2">+</td><td rowspan="2">+</td><td rowspan="2"></td><td rowspan="2">+</td></tr>
<tr><td>T</td><td>1.9</td><td>8.2</td></tr>
<tr><td rowspan="2">2</td><td>N</td><td>1.5</td><td>3.3</td><td rowspan="2">+</td><td rowspan="2">+</td><td rowspan="2">+</td><td rowspan="2">+</td><td rowspan="2">+</td><td rowspan="2"></td><td rowspan="2">+</td></tr>
<tr><td>T</td><td>1.9</td><td>3.0</td></tr>
<tr><td rowspan="2">3</td><td>N</td><td>3.9</td><td>34.9</td><td rowspan="2"></td><td rowspan="2">+</td><td rowspan="2">+</td><td rowspan="2"></td><td rowspan="2">+</td><td rowspan="2"></td><td rowspan="2"></td></tr>
<tr><td>T</td><td>2.1</td><td>31.0</td></tr>
<tr><td rowspan="2">4</td><td>N</td><td>2.1</td><td>13.4</td><td rowspan="2">+</td><td rowspan="2">+</td><td rowspan="2">+</td><td rowspan="2">+</td><td rowspan="2">+</td><td rowspan="2"></td><td rowspan="2">+</td></tr>
<tr><td>T</td><td>2.1</td><td>9.8</td></tr>
<tr><td rowspan="2">5</td><td>N</td><td>2.8</td><td>7.9</td><td rowspan="2"></td><td rowspan="2">+</td><td rowspan="2">+</td><td rowspan="2"></td><td rowspan="2">+</td><td rowspan="2"></td><td rowspan="2">+</td></tr>
<tr><td>T</td><td>3.2</td><td>45.5</td></tr>
<tr><td rowspan="2">6</td><td>N</td><td>2.1</td><td>6.7</td><td rowspan="2">+</td><td rowspan="2">+</td><td rowspan="2">+</td><td rowspan="2">+</td><td rowspan="2">+</td><td rowspan="2">+</td><td rowspan="2">+</td></tr>
<tr><td>T</td><td>1.3</td><td>7.3</td></tr>
<tr><td rowspan="2">7</td><td>N</td><td>-</td><td>3.0</td><td rowspan="2">+</td><td rowspan="2">+</td><td rowspan="2">+</td><td rowspan="2">+</td><td rowspan="2">+</td><td rowspan="2"></td><td rowspan="2">+</td></tr>
<tr><td>T</td><td>9.8</td><td>5.2</td></tr>
<tr><td rowspan="2">8</td><td>N</td><td>-</td><td>5.0</td><td rowspan="2">+</td><td rowspan="2">+</td><td rowspan="2">+</td><td rowspan="2"></td><td rowspan="2">+</td><td rowspan="2">+</td><td rowspan="2">+</td></tr>
<tr><td>T</td><td>2.5</td><td>4.4</td></tr>
<tr><td rowspan="2">9</td><td>N</td><td>2.5</td><td>6.5</td><td rowspan="2"></td><td rowspan="2">+</td><td rowspan="2">+</td><td rowspan="2">+</td><td rowspan="2">+</td><td rowspan="2"></td><td rowspan="2">+</td></tr>
<tr><td>T</td><td>2.3</td><td>17.3</td></tr>
<tr><td rowspan="2">10</td><td>N</td><td>2.4</td><td>5.7</td><td rowspan="2"></td><td rowspan="2"></td><td rowspan="2"></td><td rowspan="2"></td><td rowspan="2"></td><td rowspan="2"></td><td rowspan="2"></td></tr>
<tr><td>T</td><td>2.2</td><td>8.9</td></tr>
<tr><td rowspan="2">11</td><td>N</td><td>2.3</td><td>18.2</td><td rowspan="2"></td><td rowspan="2"></td><td rowspan="2"></td><td rowspan="2"></td><td rowspan="2"></td><td rowspan="2"></td><td rowspan="2"></td></tr>
<tr><td>T</td><td>-</td><td>1.7</td></tr>
<tr><td rowspan="2">12</td><td>N</td><td>2.1</td><td>18.2</td><td rowspan="2"></td><td rowspan="2"></td><td rowspan="2"></td><td rowspan="2"></td><td rowspan="2"></td><td rowspan="2"></td><td rowspan="2"></td></tr>
<tr><td>T</td><td>2.3</td><td>9.1</td></tr>
<tr><td rowspan="2">13</td><td>N</td><td>-</td><td>6.1</td><td rowspan="2">+</td><td rowspan="2">+</td><td rowspan="2">+</td><td rowspan="2"></td><td rowspan="2">+</td><td rowspan="2"></td><td rowspan="2">+</td></tr>
<tr><td>T</td><td>2.2</td><td>12.4</td></tr>
</table>

Table S2. Grading of tumours used in this study. The grading system has been adapted for canine simple mammary carcinomas by Clemente et al. [1] from an existing human grading system [2]. Tumours are scored from 1-3 on the level of tubule formation, nuclear pleomorphism and mitoses, giving a total score which is then converted to one of three grades; low (I), intermediate (II), or high (III).

Case#	Tumour type	Grade
1	Tubular	I
2	Cystic papillary	I
3	Tubulopapillary	I
4	Tubular	I
5	Tubular	II
6	Tubular-solid	II
7	Tubular	I
8	Tubulopapillary	I
9	Cribriform	III
10	Tubular	I
11	Tubular	II
12	Tubulopapillary	II
13	Cystic papillary	II

References

1. Clemente M, Pérez-Alenza MD, Illera JC, Peña L (2010) Histological, immunohistological, and ultrastructural description of vasculogenic mimicry in canine mammary cancer. Vet Pathol 47:265–274. doi: 10.1177/0300985809353167
2. Elston CW, Ellis IO (1991) Pathological prognostic factors in breast cancer. I. The value of histological grade in breast cancer: experience from a large study with long-term follow-up. Histopathology 19:403–410.
